# Altered Actions of Memantine and NMDA-Induced Currents in a New *Grid2-*Deleted Mouse Line

**DOI:** 10.3390/genes5041095

**Published:** 2014-12-11

**Authors:** Ayako Kumagai, Akira Fujita, Tomoki Yokoyama, Yuki Nonobe, Yasuhiro Hasaba, Tsutomu Sasaki, Yumi Itoh, Minako Koura, Osamu Suzuki, Shigeki Adachi, Haruko Ryo, Arihiro Kohara, Lokesh P. Tripathi, Masato Sanosaka, Toshiki Fukushima, Hiroyuki Takahashi, Kazuo Kitagawa, Yasuo Nagaoka, Hidehisa Kawahara, Kenji Mizuguchi, Taisei Nomura, Junichiro Matsuda, Toshihide Tabata, Hiroshi Takemori

**Affiliations:** 1 Laboratory of Cell Signaling and Metabolic Disease, National Institute of Biomedical Innovation, Saito-Asagi, Ibaraki, Osaka 567-0085, Japan; E-Mails: a-kumagai@nibio.go.jp (A.K.); y-itou@nibio.go.jp (Y.I.); m-sanosaka@nibio.go.jp (M.S.); 2 Laboratory for Neural Information Technology, Graduate School of Science and Engineering, University of Toyama, Toyama 930-8555, Japan; E-Mails: fujita0153@gmail.com (A.F.); m1371119@ems.u-toyama.ac.jp (T.Y.); weather4519@gmail.com (Y.N.); takegoshiyoshihiro45@gmail.com (Y.H.); fookun3@gmail.com (T.F.); m1471114@ems.u-toyama.ac.jp (H.T.); 3 Department of Neurology, Osaka University Graduate School of Medicine, Osaka 565-0871, Japan; E-Mails: sasaki@medone.med.osaka-u.ac.jp (T.S.); kitagawa@neurol.med.osaka-u.ac.jp (K.K.); 4 Laboratory of Animal Models for Human Diseases, National Institute of Biomedical Innovation, Saito-Asagi, Ibaraki, Osaka 567-0085, Japan; E-Mails: koura@nibio.go.jp (M.K.); osuzuki@nibio.go.jp (O.S.); jmatsuda@nibio.go.jp (J.M.); 5 Nomura Project, National Institute of Biomedical Innovation, Saito-Asagi, Ibaraki, Osaka 567-0085, Japan; E-Mails: sadachi@nibio.go.jp (S.A.); hryo@nibio.go.jp (H.R.); n5nomura@nibio.go.jp (T.N.); 6 Laboratory of Cell Cultures, National Institute of Biomedical Innovation, Saito-Asagi, Ibaraki, Osaka 567-0085, Japan; E-Mail: kohara@nibio.go.jp; 7 Laboratory of Bioinformatics, National Institute of Biomedical Innovation, Saito-Asagi, Ibaraki, Osaka 567-0085, Japan; E-Mails: lokesh@nibio.go.jp (L.P.T.); kenji@nibio.go.jp (K.M.); 8 Department of Life Science and Biotechnology, Kansai University, Suita, Osaka 564-8680, Japan; E-Mails: t010034@kansai-u.ac.jp (Y.N.); t912436@kansai-u.ac.jp (H.K.)

**Keywords:** GRID2, GluRδ2, memantine, NMDA receptor, cerebellum

## Abstract

Memantine is a non-competitive antagonist of the *N*-methyl-d-aspartate (NMDA) receptor, and is an approved drug for the treatment of moderate-to-severe Alzheimer’s disease. We identified a mouse strain with a naturally occurring mutation and an ataxic phenotype that presents with severe leg cramps. To investigate the phenotypes of these mutant mice, we screened several phenotype-modulating drugs and found that memantine (10 mg/kg) disrupted the sense of balance in the mutants. Moreover, the mutant mice showed an attenuated optokinetic response (OKR) and impaired OKR learning, which was also observed in wild-type mice treated with memantine. Microsatellite analyses indicated that the *Grid2* gene-deletion is responsible for these phenotypes. Patch-clamp analysis showed a relatively small change in NMDA-dependent current in cultured granule cells from *Grid2* gene-deleted mice, suggesting that GRID2 is important for correct NMDA receptor function. In general, NMDA receptors are activated after the activation of non-NMDA receptors, such as AMPA receptors, and AMPA receptor dysregulation also occurs in *Grid2* mutant mice. Indeed, the AMPA treatment enhanced memantine susceptibility in wild-type mice, which was indicated by balance sense and OKR impairments. The present study explores a new role for GRID2 and highlights the adverse effects of memantine in different genetic backgrounds.

## 1. Introduction

Memantine (3,5-dimethyl-1-adamantanamine) is an approved drug in the treatment of moderate-to-severe Alzheimer’s disease (AD). Ca^2+^-mediated excitotoxicity in neurons is one proposed mechanism of AD, and the *N*-methyl-d-aspartate (NMDA) receptor is one of the major receptor channels responsible for glutamate-induced Ca^2+^ influx. Memantine binds and non-competitively inhibits NMDA receptors, which subsequently protects neurons from glutamate-induced excitotoxicity [1,2].

Glutamate is essential for excitatory synaptic transmission mediated by ionotropic glutamate receptors, which include NMDA receptors and non-NMDA receptors, α-amino-3-hydroxy-5-methyl-4-isoxazolepropionic acid (AMPA) and kainate receptors [3]. NMDA receptor activation is dependent on the membrane potential that is evoked by non-NMDA receptor function [4]. Under pathological conditions, the voltage-dependent regulation of NMDA receptors is believed to be impaired, and memantine is thought to alleviate excitotoxicity by the unregulated NMDA receptor. 

Unlike memantine, other NMDA receptor antagonists, such as MK-801 [5], are considered neurotoxins because they inhibit normal excitotoxic neurotransmission as well as pathological physiological neurotransmission. Although memantine is considered a relatively safe drug, some adverse effects, such as dizziness [6], have been reported. Such adverse effects are likely to result from individual differences among patients, especially in their genetic backgrounds. However, the causes of adverse effects to memantine have not yet been clarified.

The glutamate receptor ionotropic delta 2 (GRID2, also known as GluRδ2) is abundantly expressed in cerebellar Purkinje cells, and shares sequence homology with other glutamate receptors. Despite its name and these homologies, GRID2 does not bind glutamate or other glutamate analogs [7,8,9]. Mice with an impaired *Grid2* gene exhibit a broad range of phenotypes, such as cerebellar ataxia, poor motor learning, and memory dysfunction. In addition to the known phenotypes in mice, new phenotypes presumably involving NMDA receptor dysfunction or memantine effects, such as nystagmus (in frogs) [10], oculomotor apraxia (in cats) [11,12], dementia (in humans) [13,14], have been observed in human patients with *GRID2* gene deletions [15,16,17,18]. However, there is not sufficient evidence to ascribe these complex symptoms in human patients to *GRID2* gene deletions.

Here, we report a new deletion in the mouse *Grid2* gene that is accompanied by ataxia. Administration of memantine led to impaired balance in ataxic mice, and the mutant mice showed deficits in the optokinetic response (OKR) and its learning. These optokinetic impairments were also sensitive to memantine. In addition, only a small population of cultured granule cells isolated from the mutant mice showed memantine-sensitive NMDA-induced currents. These phenomena were mimicked in wild-type (WT) mice following co-treatment with memantine and AMPA.

## 2. Results 

### 2.1. The Hereditary Ataxic Mouse is Sensitive to Memantine

**Figure 1:**
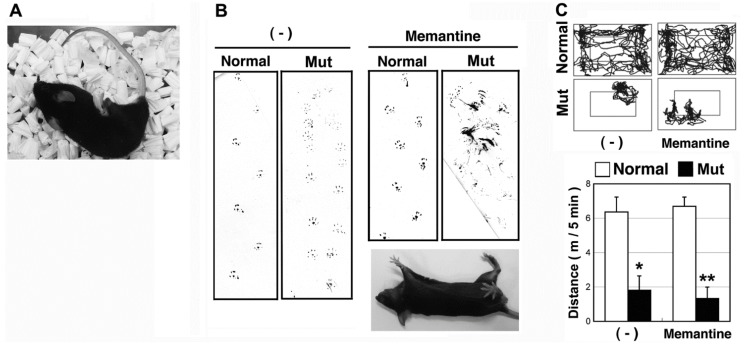
Isolation of ataxic mice with memantine susceptibility. (**A**) A mouse (6-month-old female) that exhibited an ataxic phenotype with rigid hind limbs. The phenotype became more severe with age; (**B**) Footprint analyses 10 min after memantine treatment (10 mg/kg, 12-week-old male). The soles of the hind limbs were labeled with India ink for a mouse that walked freely on paper. Rollover by the mutant mouse after memantine treatment is shown (lower right); (**C**) Monitoring mice walking after memantine treatment (10 mg/kg, 12-week-old male). The position of the mouse’s head was tracked (left), and the walking distance was recorded for 5 min (right; expressed as the mean and SD; n = 6). * *p* < 0.05, ** *p* < 0.01.

Two ataxic (male and female) mice appeared spontaneously in the same litter from a mating pair of hetero mice with the knockout (KO) allele for Salt-Inducible Kinase 3 (*Sik3*: *Sik3^tm1Htake/+^*) [19] on a C57BL/6J (B6) genetic background. The male was *Sik3^+/−^*, but the female was *Sik3^+/+^*. Because these ataxic mice failed to produce offspring by natural mating, we performed *in vitro* fertilization (IVF) using these ataxic mice and confirmed the heredity of the phenotype. To expound the mouse population, the original sperm of the ataxic male was also used for other IVF with the oocyte of normal B6 female, and offspring with the ataxic phenotype were obtained at the F2 generation (numbers of normal male, ataxic male, normal female, and ataxic female were 4, 1, 9, and 2, respectively), suggesting a recessively-inherited phenotype. Despite showing normal forelimb movements, the mutant offspring were characterized by a short-stepped gait and frequent falls due to suspected leg cramps (Figure 1A).

To examine whether the phenotype was caused by a neurogenic disorder, neuropharmacological compounds were tested in the normal and mutant mice. Because anesthesia (1% isoflurane) [20] alleviated leg cramps in the mutant mice, we first selected anticonvulsant drugs including gamma-aminobutyric acid (GABA) receptor activators and NMDA receptor antagonists (Table 1). The GABA-A receptor activators felbamate and nitrazepam failed to modulate the phenotypes in the mutant mice. Whereas, memantine (10 mg/kg), an NMDA and 5HT_3_ receptor antagonist which is also reported to exert agonistic actions for the dopamine D2 receptor [21,22], impaired balance in the mutant mice, but not normal mice (Figure 1B and Supplementary Movie 1). Movement traces of the mice (Figure 1C) confirmed that 10 mg/kg memantine had no significant effect on walking in normal mice.

Next, we tested other NMDA receptor modulators. Similar to memantine, the NMDA receptor antagonist MK-801 (10 mg/kg) led to balance disturbances in the mutant mice. However, the normal mice also showed balance disturbances at this dose (Table 1). (*R*)-CPP also produced balance disturbances only in the mutant mice (Supplementary Movie 2), whereas ifenprodil, Ro25-6981, or the AMPA receptor antagonist DNQX did not produce balance disturbances in the mutant or normal mice. However, slower movements were observed in mutant mice following administration of DL-AP7. 

Because serotonin reuptake inhibitors are used to treat vertigo [23], and the 5HT_3_ antagonist ondansetron causes headache and dizziness [24], we examined whether ondansetron affected the mutant mice. Both mutant and normal mice showed decreased activity with ondansetron (10 mg/kg; Supplementary Movie 3), but no effect that was specific to mutant mice was observed. Moreover, enhanced cholinergic signaling following donepezil treatment [21] and activation of dopamine signals by L-Dopa [21,22] had no significant effect on behavior in both normal and mutant mice (Table 1). These results suggest that, in addition to the ataxic phenotype, the mutant mice were characterized by enhanced memantine susceptibility, which was probably due to impaired NMDA receptor functions. The inconsistency in the efficacy of NMDA receptor antagonists on balance disturbance might result from differences in NMDA receptor subunit subtype-specificity and/or effective dose among the antagonists; 10 mg/kg and 10–20 mg/kg might be the threshold doses to affect balance in the mutant mice for memantine and (*R*)-CPP, respectively.

**Table 1:** Pharmacological effectors on mutant mice phenotypes.

Chemical	Category	Dose (mg/kg)	Number of Mice	Movement of Normal Mice	Movement of Mutant Mice
Memantine	NMDA-R antagonist	2 10	2 2	NS NS	NS Balance loss
MK-801	NMDA-R antagonist	2 10	2 3	NS Balance loss	NS Balance loss
DL-AP7	NMDA-R antagonist	30	2	NS	Slow movement
(R)-CPP	NMDA-R antagonist (NR2A antagonist)	10 20	4 2	NS NS	Balance loss * Balance loss
Ro25-6981	NMDA-R antagonist	2 10	3 3	NS NS	NS NS
Ifenprodil	NMDA-R antagonist (NR2B antagonist)	2 10	3 3	NS NS	NS NS
Felbamate	NR2B antagonist GABA-R activator	30	2	NS	NS
Nitrazepam	GABA-R activator	30	2	NS	NS
Isoflurane	GABA-R activator	1% in air	2	Slip	Slip
DNQX	AMPA/Kainate receptor antagonist	2 10	3 3	NS NS	NS NS
Ondansetron	5-HT3 antagonist	5 10	2 2	NS Not move	NS Not move
Donepezil	acetylcholinesterase inhibitor	10	3	NS	NS
l-Dopa	Dopamine precursor	20	3	NS	NS

NS: No significant effect was observed. *: 2 mice.

### 2.2. Impaired OKR and Learning in the Ataxic Mice

Gait disorders are a common pathology in patients with spinocerebellar degeneration, and are sometimes accompanied by vertigo resulting from oculomotor dysfunction [25,26]. Memantine also affects oculomotor functions in patients with cerebellar ataxia [27]. To examine whether our mutant mice also had oculomotor impairments, we measured the OKR and OKR adaptation, a form of cerebellum-dependent learning [28,29], and used the results from these assays to quantify memantine susceptibility (Supplementary Figure S1).

**Figure 2:**
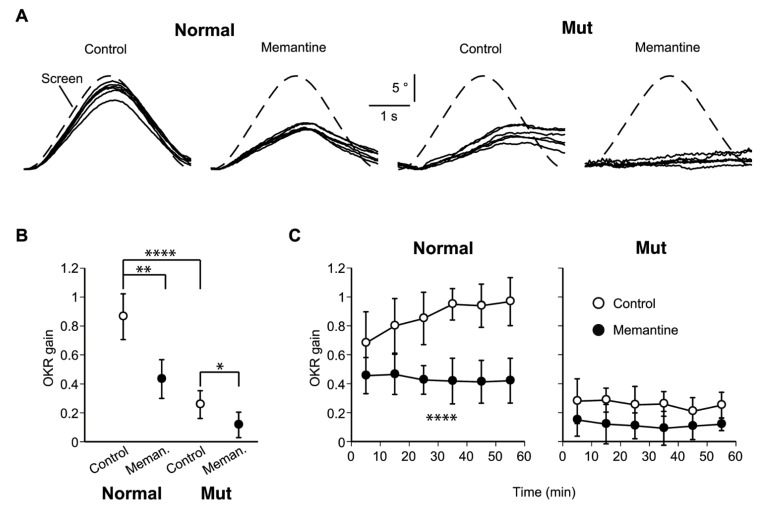
The optokinetic response (OKR) and its susceptibility to memantine. (**A**) The OKR of normal and mutant mice after intraperitoneal injection of saline (control) or memantine-containing saline (10 mg/kg). Measurements commenced 10 min after injection. Representative OKRs from normal and mutant mice are shown. The relative pupil azimuth is plotted against time. Each trace indicates the average over each 10-min period of a 1-h measurement session. Screen, movement of the stimulus screen; (**B**) Overall OKR gain throughout the 1-h session with or without memantine. Dots and error bars, mean ± SD; n = 5 for each data point. * *p <* 0.05, ** *p <* 0.01, and **** *p <* 0.0001, multivariate ANOVA. The overall OKR gain was calculated by averaging the OKR gains for all 10-min periods in a single 1-h session (see panel C); (**C**) Time course of the mean OKR gain for normal (n = 5) and mutant (n = 5) mice. Each dot indicates the average over a 10-min period of a 1-h measurement session. Error bars indicate ±SD. **** *p* < 0.0001 *vs.* the control for time × drug interaction, repeated measures ANOVA.

Normal and mutant mice were subjected to the OKR assay and horizontal visual pattern oscillations were given (Figure 2A). The normal mice could track the stimulus screen, whereas the mutant mice could not. Memantine (10 mg/kg) significantly lowered the overall OKR gain throughout a 1 h session in normal mice while the OKR in mutant mice was completely abolished (Figure 2B).

To evaluate OKR adaptation, the mean OKR gain was plotted for each 10-min interval (Figure 2C). Saline-injected normal mice (control) exhibited a time-dependent increase in the OKR gain, but no gain increase was observed in the mutant or the memantine-treated normal mice. 

### 2.3. Microsatellite Analysis in the Ataxic Mice

**Figure 3:**
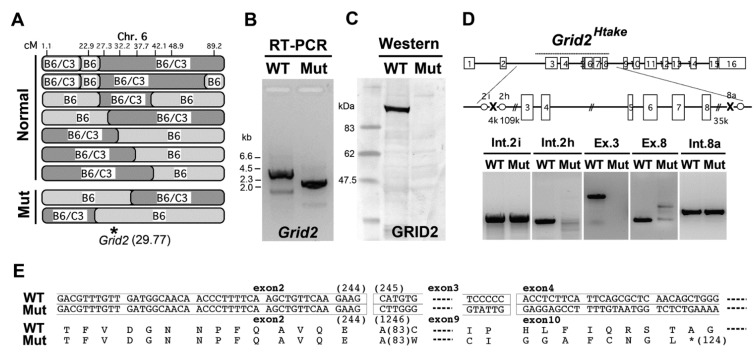
Identification of the responsible gene. (**A**) Microsatellite analyses of N2 mice. B6, parental allele of the ataxic mouse (C56BL/6J); C3, WT allele of C3H/HeN. N2 mice were produced following *in vitro* fertilization with oocytes from hetero N1 (B6/C3) and sperm from the original N0 ataxic B6 (B6/B6). Markers are described in the Experimental Section. Normal and Mut indicate mice with or without the ataxic phenotype, respectively. The *Grid2* gene is located 29.77 cM in chromosome 6; (**B**) The full-length open reading frame ORF of *Grid2* was amplified from cDNA prepared from the cerebellum of WT and Mut mice; (**C**) Western blotting for GRID2; (**D**) A diagram showing break points in the *Grid2* gene of ataxic mice; (**E**) Putative protein sequence of GRID2 in *Grid2^Htake/Htake^* mice is predicted from diagram D and the direct ORF sequence.

Because the pharmacological analyses were unable to predict the gene responsible for the ataxic phenotype, we performed a microsatellite analysis to identify the gene. Sperm were isolated from an ataxic mouse (N0: C57BL/6J[B6], B6/B6) and used to perform IVF with oocytes from C3H/HeN (C3) mice (normal: C3/C3). Oocytes were prepared from the N1 female (B6/C3 hetero-mice) and used for a second round of IVF using the original sperm from the N0 (B6/B6) ataxic mice, which produced 137 N2 mice (75 non-ataxic (the responsible genomic-region should be B6/C3) and 62 ataxic (the responsible genomic-region should be B6/B6)). Using 60 microsatellite markers and DNA from eight N2 non-ataxic mice, we screened heterogenic-regions composed of both B6 and C3 chromosomes. We observed that D6Mit149 (Chromosome 6, 48.93 cM) was amplified as the heterogenic-type in all eight mice. Next, we analyzed other markers near D6Mit149 using DNA from 70 mice (Figure 3A) and narrowed the responsible region to 27.3–32.2 cM (D6Mit384–D6Mit243), which was also associated with the phenotype of enhanced memantine susceptibility.

When we searched for genes that have known associations with ataxic phenotypes, we noticed the *Grid2* gene at 29.77 cM [30]. To determine whether this gene was responsible for the observed phenotype, the *Grid2* open reading frame (ORF) was amplified from cDNA prepared from the cerebellum. As shown in Figure 3B, the *Grid2* ORF was approximately 1 kb shorter in the mutant mice than in the WT mice. In addition, western blotting for GRID2 revealed that this protein was absent from the mutant cerebellum (Figure 3C). 

Direct sequencing of the mutant ORF suggested that the region from exon 3 to exon 8 might be deleted, which is the longest deletion reported in *Grid2* mutant mice [31]. Chromosome walking (Figure 3D) identified the break points 110 kb upstream from exon 3 and 30 kb downstream from exon 8, which yielded a truncated GRID2 protein similar to that in the *Grid2^trp/trp^*, *Grid2^ho8J/ho8J^*, and *Grid2^ho13J/ho13J^* mutants, but with different flanking peptides (Figure 3E). Our laboratory code for mutant mouse lines is Htake, thus the mutant allele is named *Grid2^Htake^*. 

### 2.4. Altered Sensitivity to NMDA in Cultured Granule Cells of Grid2^Htake/Htake^ Mice

**Figure 4:**
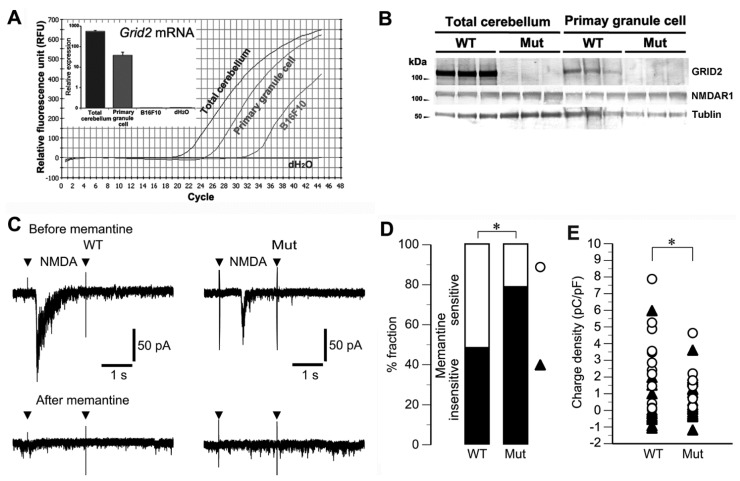
NMDA-responsiveness of cultured cerebellar granule cells. (**A**) The raw data for quantitative PCR analyses and normalized levels (inset: n = 4) of *Gird2* mRNA are indicated. Total RNA was prepared from total cerebellum, 2-week-cultured cerebellar granule cells from neonates (P2), B16F10 melanoma cells (non-neuronal negative control); (**B**) Western blotting was performed using WT and *Grid2^Htake/Htake^* (Mut) total cerebella or cultured granule cells. There is no significant difference in NMDAR1 protein level; (**C**) Whole-cell current responses of cultured granule cells derived from WT and Mut mice to local application of NMDA (20 µM, 2 s) before and after treatment with memantine (10 µM, 30 s). Representative currents recorded from single WT and Mut cells. Arrowheads indicate artifacts due to the opening and closure of the electromagnetic valve controlling delivery of NMDA-containing saline; (**D**) The examined cells were categorized into two groups by memantine susceptibility, and their populations are represented as % fractions. When more than 75% of NMDA-induced inward current was suppressed by memantine, the cells were categorized into the memantine-sensitive group (white area). Cells that experienced reduced inward current suppression after memantine treatment, or no significant inward current were categorized into the memantine-insensitive group (black area). * *p* = 0.0178, likelihood ratio test (WT, n = 27; Mut, n = 28); (**E**) Distribution of the total charge density of NMDA-induced inward currents. Open circles (white) and closed triangles (black) indicate the data from cells categorized as D. The magnitude of the total data (white and black) was not significantly different between the WT and mutant cells (medians, 1.01 and 0.64 pC/pF, respectively). However, in a comparison of the data from the memantine-sensitive cells (white), there was a significant difference in variance between the WT and mutant cells (* *p* = 0.0120, Brown-Forsythe test).

GRID2 is highly expressed in Purkinje cells, and disruption of GRID2 signaling impairs Purkinje cell functions. However, little or no expression of functional NMDA receptors in Purkinje cells of the adult cerebellum has been reported [32]. Thus, it is possible that *Grid2* gene deletion influences cerebellar function by affecting NMDA receptors on granule cells. We examined whether *Grid2^Htake/Htake^* mice possessed memantine-sensitive NMDA receptors on cerebellar granule cells, using granule cell-enriched (Purkinje cell-free) primary cultures from P2 mice. Quantitative PCR (Figure 4A) and western blot analyses (Figure 4B) showed that cultured granule cells expressed low, but detectable, levels of *Grid2* mRNA and GRID2 protein. No significant difference in NMDAR1 protein level was observed between the cultures derived from normal and *Grid2^Htake/Htake^* mice, which was consistent with the result of mRNA (microarray) analyses (Supplementary Table S1).

We have to note, however, that no significant interaction between GRID2 protein and NMDAR1 protein in the total cerebella or cultured granule cell lysate was observed (Supplementary Figure S2), despite the presence of PKC gamma binding to GRID2 protein [33].

Next, we monitored NMDA-induced currents in cultured granule cells using a whole-cell voltage-clamp technique. At cell densities as low as 1.25 million cells/mL and a holding potential of −90 mV, granule cells from both WT and mutant mice showed no baseline activity (Figure 4C). In the absence of memantine (before memantine), NMDA (20 µM, 2 s) induced inward currents that were larger in WT cells than in mutant cells. Furthermore, NMDA-induced currents were reduced in the presence of memantine (after memantine: 10 µM, 30 s). However, the magnitude of the memantine-dependent suppression of the NMDA-induced currents varied in the individual cells.

To accurately categorize the memantine-sensitive NMDA-induced currents, we divided the cells into two categories based on the ability of memantine to reduce the charge density of NMDA-induced currents by more than 75%. According to this classification, 51.9% of WT cells and only 21.4% of mutant cells were categorized as memantine-sensitive (Figure 4D). Moreover, the classification (only open circles) revealed that the values of charge density from the mutant cells fell into a lower range, which produced a significant difference in the variance of the charge density between the WT and mutant groups (Figure 4E). These results suggest that mutant mice might possess a poor variation of NMDA receptor with reduced function on their granule cell population. 

### 2.5. Mice Treated with Memantine and AMPA Were Unable to Walk Smoothly

**Figure 5:**
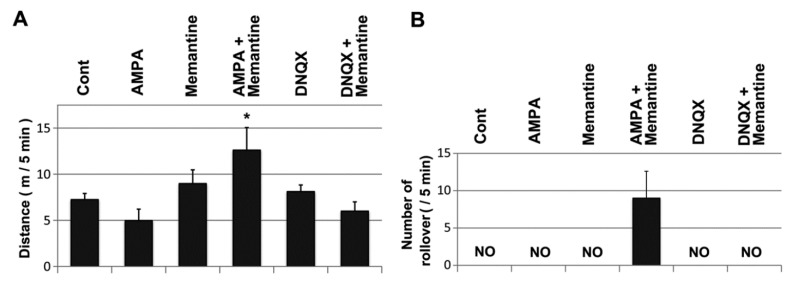

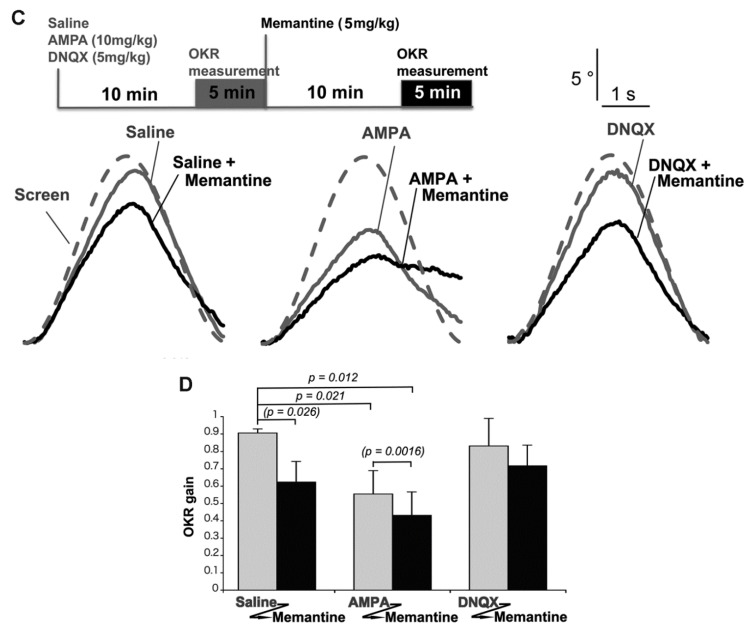
Effect of AMPA receptor modulators on memantine action in WT mice. (**A**) Monitoring of walking mice (12-week-old WT male, n = 6) after memantine treatment (10 mg/kg) combined with AMPA (20 mg/kg) or the AMPA receptor antagonist DNQX (10 mg/kg). Ten minutes after the treatments, the walking distance for 5 min was expressed as the mean and SD, * *p* < 0.05 *vs.* the other conditions (unpaired *t*-test; *p* < 0.0001 for overall differences, one-way ANOVA); (**B**) Number of rollovers in 5 min was counted. NO indicates that rollover was not observed. n = 6 for Control and AMPA + memantine and n = 3 for the other conditions. The occurrence of rollover was significantly different between AMPA + memantine and the other conditions (*p* < 0.05, Fisher’s exact test); (**C**) OKRs measured in female WT mice with sequential injections of the control saline, AMPA (10 mg/kg), or DNQX (5 mg/kg) and memantine (5 mg/kg). A set of traces indicates representative responses of individual mice. Dotted line, the movement of the stimulus screen; (**D**) Graph shows the mean and ±SD of OKR gain (n = 3 for each condition). *p* value (without a bracket, one-tailed unpaired *t*-test; with brackets, paired *t*-test) is indicated when significance was observed.

GRID2 deficiency results in dysregulation of AMPA receptors [34,35]. To examine whether impaired AMPA receptor functions affected memantine susceptibility, mice were treated with memantine simultaneously with the AMPA receptor agonist AMPA or the antagonist DNQX [36], and the movements of these mice were monitored (Figure 5A). The mice treated with AMPA (20 mg/kg) walked slowly and sometimes crouched on the floor. However, mice treated with both memantine (10 mg/kg) and AMPA had increased activity and did not stop walking. In addition to these abnormal behaviors, the mice walked with a mild staggering gait and sometimes slipped (roll-over: Figure 5B, Supplementary Movie 4). These combined effects of memantine on mouse behavior were not observed when memantine was administered with DNQX (10 mg/kg), although the mice treated with DNQX were also sometimes crouched. Moreover, in the *Grid2^Htake/Htake^* mice, co-treatment with AMPA and a low dose of memantine (5 mg/kg) caused more evident balance impairment than memantine treatment alone (Supplementary Figure S3), suggesting that GRID2 deficiency may augment the synergistic action of AMPA and memantine. The effect of AMPA co-treatment could not be evaluated with a higher dose of memantine (10 mg/kg) because the maximal effect was induced by this dose of memantine.

Finally, the OKR was monitored after co-treatment with memantine and AMPA or DNQX (Figure 5C). Mice were first treated with AMPA, DNQX, or saline, and the OKR was subsequently monitored for 5 min (because some mice closed their eyes after treatment with higher doses, AMPA (10 mg/kg) and DNQX (5 m/kg) were used for OKR measurement). To evaluate synergies between memantine and AMPA receptor modulators, the mice were further treated with a low dose of memantine (5 mg/kg), and again subjected to OKR measurements for another 5 min. AMPA significantly impaired the OKR, and the combined treatment with memantine further impaired the OKR (Figure 5D). These effects were not observed in mice co-treated with memantine and DNQX. 

## 3. Discussion

Here, we report cross-talk between GRID2 signaling and memantine in mice, which may, in part, account for the adverse effects of memantine in patients with individual differences in congenital or acquired genetic factors, such as GRID2.

Major phenotypes have been identified in GRID2 mutant mice, including impaired motor coordination, learning, and memory. GRID2 is located on the postsynaptic membrane of Purkinje cells and binds to cerebellin precursor protein 1 (CBLN1) and neurexin 1 beta (NRXN1b) [7,8] on the parallel fibers of granule cells [7,37]. On the other hand, the memantine target, NMDA receptors, are also expressed on the developing [38] and adult [39] cerebellar granule cells. Furthermore, contrary to the neurotoxic effects of glutamate, NMDA receptors were found to promote the survival of cultured Purkinje [40] and granule cells [41,42]. However, double-KO mice with disrupted *Nr2A* and *Nr2C* genes (encoding two major NMDA receptor subunits in the adult mice cerebellum) demonstrate a mild impairment in motor coordination, but they do not exhibit an ataxic phenotype [43]. This observation suggests that NMDA receptors and GRID2 functions may not be directly linked [44].

Thus, in the present study, pharmaco-behavioral approaches failed to identify *Grid2* as a candidate for the cause of the observed phenotypes. Unexpectedly, our efforts resulted in the observation of new phenotypes in our *Grid2* deficient mice, which appeared as enhanced memantine susceptibility, which was likely mediated by dysfunctional NMDA receptors [1,2,45]. OKR measurements in *Grid2^Htake/Htake^* mice revealed impaired basal cerebellar functions in eye movement, which was also mimicked by memantine treatment. Dizziness has been reported as a major adverse effect of memantine treatment in humans [6,46]. The cerebellar flocculus is thought to be responsible for the early stage formation of OKR adaptation and its memory [47,48]. 

The *Grid2* gene is located in a hot spot of genomic deletions [31], and a number of mutant lines with defects in this gene have been identified. In addition to naturally occurring mutants, targeted disruption and knock-in mutations of *Grid2* have been reported [30,49,50]. In contrast to these loss-of-function mutations, *Grid2^Lc^* (*Lurcher*) [51] was identified as a spontaneous dominant mutation characterized by cerebellar ataxia and atrophy of Purkinje and granule cells [30]. Physiological studies of *Grid2^Lc/+^* mice have shown that the *Grid2^Lc^* mutation produces constitutive inward Ca^2+^/Na^+^ currents that induce cell death [52].

The importance of the genetic background on *Grid2^Lc/+^* mice phenotypes was also reported [47,53]. In congenic *Grid2^Lc/+^* mice, almost 99.99% of those on a C57BL/6 genetic background lost Purkinje cells, whereas no Purkinje cell loss was observed in *Grid2^Lc/+^* mice on a 93% C57BL/6 genetic background, indicating that phenotypes in *Grid2^Lc/+^* mice are highly dependent on their genetic backgrounds. Interestingly, abnormal eye-movement and impaired motor-coordination were only observed in 93% of C57BL/6 background-*Grid2^Lc/+^* mice possessing Purkinje cells, but not in 99.99% of C57BL/6 background*-Grid2^Lc/+^* mice without Purkinje cells [47], suggesting that gain of GRID2 signaling is also a cause of motor deficits in the presence of Purkinje cells. In contrast to *Grid2^Lc/+^* mice, we noticed during gene mapping that memantine-induced balance impairment was observed in *Grid2^Htake/Htake^* mice irrespective of their genetic backgrounds (mixed B6 and C3 backgrounds). This is also the case for random eye movements commonly observed in different *Grid2*-deleted mice with different genetic backgrounds, *Grid2*-KO in C57BL/6 and *Grid2^ho-15J/15J^* on a C3HJ background [17], suggested that memantine-induced balance impairment may occur in other *Grid2* deficient mice irrespective of their genetic backgrounds. 

How does the *Grid2^Htake^* deletion enhance the actions of memantine? GRID2 regulates long-term depression (LTD) at synapses between immature parallel fibers and Purkinje cells by inducing AMPA receptor endocytosis [34,35]. d-Serine is an endogenous ligand for GRID2 and one of the factors that induce LTD [9]. Developing mice that express GRID2 with a disrupted d-serine binding site show impaired motor coordination and learning, suggesting the importance of LTD for motor regulation. On the other hand, NMDA receptor activation requires the removal of Mg^2+^ block, which occurs when the membrane potential increases through activation of non-NMDA receptors including AMPA receptors [4]. Cooperative signal-transmission from cerebellar mossy fiber-granule cells to Purkinje cells mediated by NMDA and AMPA receptors has been shown in both Mg^2+^ block dependent and independent manners [54]. We observed that co-treatment with memantine and AMPA impaired gait and OKR in wild type mice, suggesting that dysregulation of AMPA receptor function in *Grid2* deficient mice may cause the enhanced memantine susceptibility. This may be implicated in the decreased number of memantine-sensitive NMDA-responsible granule cells in *Grid2^Htake/Htake^* mice. 

Whilst mapping the gene responsible for the ataxic phenotype, we observed new phenotypes in *Grid2* deficient mice, which were latent balance defects, and OKR impairments. These deficits were also mimicked by memantine with AMPA in the impaired WT mice. Because, recently, the wide distributions of *Grid2* mRNA and GRID2 protein were reported in the adult rodent brain [55], the phenotypes in *Grid2^Htake/Htake^* mice could be ascribed to not only attenuated NMDA receptor responsiveness in a subset of granule cells but also other cellular mechanisms. 

When granule cells are collectively activated *in vivo*, glutamate spilt over from parallel fiber-Purkinje cell synapses stimulate adjacent interneurons (volume transmission), which in turn exert inhibition of Purkinje cells [44,56,57,58] crucial for normal motor coordination [59]. The GRID2 mutation may reduce NMDA receptor-mediated excitation of granule cells, and this might decrease the volume transmission and Purkinje cell inhibition via interneurons. Moreover, cerebellar interneurons (basket, stellate, and Golgi cells) also express functional NMDA receptors at their presynaptic membrane [44,56,57,58] and these receptors could be affected by the change of GRID2 signaling. Taken as a whole, the present study using a naturally occurring *Grid2* deleted mouse line may lead to a better understanding of NMDA, AMPA, and GRID2 receptors. Further studies are required to elucidate the precise mechanisms underlying GRID2 signaling.

## 4. Experimental Section

### 4.1. Animals

C57BL/6J (B6) and C3H/HeN (C3) from SLC Japan (Shizuoka, JAPAN) were used for the maintenance of the *Grid2^Htake/Htake^* mouse line and for IVF for microsatellite analyses, respectively. IVF was performed following a standard method [60] using human tubal fluid medium (Ark Resource, Kumamoto, Japan). The *Grid2* mutant mice, *Grid2^Htake/Htake^* (formal name is *Grid2^ho-Htake^*/Nibio), were supplied by the JCRB Laboratory Animal Resource Bank at the National Institute of Biomedical Innovation. The experimental mouse protocols were approved by the Ethics Committee at the National Institute of Biomedical Innovation (assigned No. DS-23-35), and by the University of Toyama’s Committee on Animal Experiments (assigned No. A2012eng-8) for animal welfare. For microarray analyses, cerebella were dissected after anesthesia of mice with isoflurane (WAKO Pure Chemicals, Osaka, JAPAN). The animals were maintained under standard light (08:00–20:00) and temperature conditions (23 °C, 50% humidity).

### 4.2. Reagents

Microsatellite markers were used to identify the chromosomal region responsible for the ataxic phenotype, (D6Mit86, 1.18 cM; D6Mit351, 22.94 cM; D6Mit384, 27.38 cM; D6Mit243, 32.2 cM 7; D6Mit29, 37.75 cM; D6Mit102. 42.11 cM; D6Mit149, 48.93 cM; D6Mit200, 89.28 cM). To map the end points of the *Grid2* deletion, we used following primers: m*Grid2* intron 2H F 5'-GCT ACT TTG GTA CAA GTG GAC A and m*Grid2* intron 2H R 5'-GAC AAG TTG CTC TCT GTA TCT; m*Grid2* Intron 2I F 5'-CAT GCT CAC ATC AAA ATA CAT CAA and m*Grid2* Intron 2I R 5'-TGT AAT TGA GGAA AAT ACA TAA T. Other primers used in Figure 3D were prepared with reference to [31]. To identify the *Grid2* deletion in *Grid2^Htake/Htake^* mice, we used the following primers for PCR amplification: m*Grid2* Intron 2HIF 5'-TGG ATC CTT CTA CGT GCA AC, m*Grid2* Intron 8YF2 5'-GGA CCA CAC TGA GGT TCG AAA GA, and m*Grid2* Intron 8YR 5'-ATC TCT TGG CAT GCA TTA GAC. The mutant allele of *Grid2^Htake^* produces a PCR band corresponding to a product of approximately 600 bp, and that for the WT allele is approximately 400 bp.

GRID2 antibody was obtained from Santa Cruz Biotechnology (Santa Cruz, CA, USA). Antibodies for NMDAR1 and tubulin were obtained from Cell Signaling Technology (Boston, MA, USA). Memantine, AMPA, DNQX, MK-801, nitrazepam, donepezil, and ondansetron were purchased from WAKO Pure Chemicals; ifenprodil, Ro25-6981, DL-AP7, felbamate, and loperamide were obtained from Sigma-Aldrich (St. Louis, MO, USA).

### 4.3. mRNA Analyses

Total RNA was prepared from the cerebellar hemispheres of 12-week-old mice (male and female) using the EZ1-RNA purification kit (Qiagen, Venlo Park, The Netherlands). For microarray analyses, GeneChip Mouse Genome 430A (Affymetrix, Santa Clara, CA, USA) was used, and differences in transcript levels were calculated with Partek (Partek Inc., St. Louis, MO, USA). The original data files (CEL-files) were deposited in the Gene Expression Omnibus (GEO) repository and assigned the GEO accession numbers: GSM1334015, GSM1334016, and GSM1334017 for normal mice, and GSM1334018, GSM1334019, and GSM1334020 for mutant mice. PCR primers for real-time PCR were as follows: *mGrid2* F, 5'-AAC ACG CTA CAT GGA CTA CTC-3' and *mGrid2* R, 5'-GAA GCA CTG TGC CAG CAA TG-3'; m*Gapdh* F, 5'-ACT CAC GGC AAA TTC AAC GG-3' and m*Gapdh* R, 5'-GAC TCC ACG ACA TAC TGA GC-3'. To amplify the *Grid2* ORF, primers are mGrid2 F2 (5ATG): 5'-ATG GAA GTT TTC CCC TTG CTC TTG T and mGrid2 R (3Stop): 5'-TCA TAT GGA CGT GCC TCG GTC GGG GTC A were used.

### 4.4. Measurements of Walking Distance and the OKR

Male mice (12–14 weeks old) were placed in a rectangular box (25 cm × 40 cm), and their head position was tracked for 5 min using ANY-maze software (Brain Science Idea, Osaka, Japan). Each mouse was monitored three times, and the longest distance recorded was used in the data analysis.

The OKR was measured in adult mice using previously described methods [28], which are depicted in Supplementary Figure S1. Briefly, the mice were anesthetized with isoflurane (2%) and a stainless steel screw was glued to the skull. The mice were then habituated to the experimental conditions for 2 days before the measurement. Ten minutes before the measurement were made, mice were administered a saline (8 mL/kg) injection with or without memantine (10 mg/kg) intraperitoneally. The mouse was then mounted on a stereotaxic apparatus and exposed to continuous sinusoidal horizontal oscillations (17°, 0.25 Hz) of a cylindrical checkerboard-patterned screen (diameter, 65 cm; single square, 1.8 × 1.8 cm; brightness, ~30 lx). Right eye movement was captured at 30 Hz with an infrared camera. For each image frame, we used a machine vision system to estimate the pupil azimuth from the location of the pupil center. The OKR was expressed on a time plot of the relative pupil azimuth for each round of screen oscillation, with the pupil azimuth at the beginning of backward eye movement set to 0°. An OKR gain was defined as the ratio of the maximal relative pupil azimuth change to that of the screen (17°).

### 4.5. Cell Culture and Electrophysiology

Granule cell-enriched cultures were prepared as previously described [61]. Briefly, 2-day-old (P2) mutant or WT mice were anesthetized by cooling and then sacrificed by decapitation. The cerebella were dissociated with trypsin, plated on poly-l-ornithine-coated plastic dishes (Becton Dickinson, Franklin Lakes, NJ, USA) at 1.25 million cells/mL, and maintained in low-serum, nutrient-supplemented Dulbecco’s Modified Eagle Medium/F-12 (Life Technologies; 5% CO_2_, 37 °C) for 12 days.

Ruptured-patch whole-cell recordings were performed on cultured granule cells. The pipette solution contained 134 mM potassium d-gluconic acid, 7.6 mM KCl, 9 mM KOH, 10 mM NaCl, 1.2 mM MgCl_2_, 4 mM ATP magnesium salt, 10 mM HEPES, and 0.5 mM EGTA (pH 7.3). The culture dish was perfused at a rate of 1.4 mL/min with 145 mM NaCl, 5 mM KCl, 2 mM CaCl_2_, 10 mM HEPES, 10 mM d-glucose, and 10 µM glycine (pH 7.4). Current signals were recorded using an EPC-8 amplifier (holding potential, −90 mV; cut-off frequency, 5 kHz; sampling rate, 20 kHz; HEKA, Lambrecht/Pfalz, Germany) controlled by Patchmaster software (version, 2.35; HEKA). The command potentials were corrected for a liquid junction potential between the pipette and bath solutions. Electronic capacitance cancellation and series resistance compensation were not used. The series resistance (33.2 ± 5.1 MΩ, n = 55) and membrane capacitance were estimated from the amplitude and time constant of the capacitive current evoked by a 10 mV voltage jump. The bath solution containing 20 µM NMDA or 10 µM memantine was locally applied to the cell through a theta tube under the control of gravity and electromagnetic valves (VM8, ALA Scientific Instruments, Farmingdale, NY, USA). The magnitude of an NMDA-induced current was quantified as the inward current charge over a 2 s NMDA application normalized to the membrane capacitance (charge density). The background charge was estimated from a 0.5 s pre-application period and subtracted from the charge density.

### 4.6. Statistical Analyses

Data from each group were characterized by the mean ± SD, unless otherwise stated. Data from biochemical assays were examined with one-way ANOVA followed by unpaired two-tailed *t*-tests to detect statistically significant differences. All the statistical examinations were performed using JMP software (versions 9.0.2 and 10.0.1, SAS Institute, Cary, NC, USA).

## Supplementary Files

Supplementary File 1

## References

[1] Sonkusare S.K., Kaul C.L., Ramarao P. (2005). Dementia of Alzheimer’s disease and other neurodegenerative disorders—Memantine, a new hope. Pharmacol. Res..

[2] Lipton S.A. (2006). Paradigm shift in neuroprotection by NMDA receptor blockade: Memantine and beyond. Nat. Rev. Drug Discov..

[3] Szczurowska E., Mares P. (2013). NMDA and AMPA receptors: Development and status epilepticus. Physiol. Res..

[4] Kupper J., Ascher P., Neyton J. (1996). Probing the pore region of recombinant N-methyl-d-aspartate channels using external and internal magnesium block. Proc. Natl. Acad. Sci. USA.

[5] Wong E.H., Kemp J.A., Priestley T., Knight A.R., Woodruff G.N., Iversen L.L. (1986). The anticonvulsant MK-801 is a potent N-methyl-d-aspartate antagonist. Proc. Natl. Acad. Sci. USA.

[6] Hansen R.A., Gartlehner G., Lohr K.N., Kaufer D.I. (2007). Functional outcomes of drug treatment in Alzheimer’s disease: A systematic review and meta-analysis. Drugs Aging.

[7] Uemura T., Lee S.J., Yasumura M., Takeuchi T., Yoshida T., Ra M., Taguchi R., Sakimura K., Mishina M. (2010). Trans-synaptic interaction of GluRdelta2 and Neurexin through Cbln1 mediates synapse formation in the cerebellum. Cell.

[8] Matsuda K., Miura E., Miyazaki T., Kakegawa W., Emi K., Narumi S., Fukazawa Y., Ito-Ishida A., Kondo T., Shigemoto R. (2010). Cbln1 is a ligand for an orphan glutamate receptor delta2, a bidirectional synapse organizer. Science.

[9] Kakegawa W., Miyoshi Y., Hamase K., Matsuda S., Matsuda K., Kohda K., Emi K., Motohashi J., Konno R., Zaitsu K. (2011). d-Serine regulates cerebellar LTD and motor coordination through the delta2 glutamate receptor. Nat. Neurosci..

[10] Jardon B., Bonaventure N. (1992). N-Methyl-d-aspartate antagonists suppress the development of frog symmetric monocular optokynetic nystagmus observed after unilateral visual deprivation. Brain Res. Dev. Brain Res..

[11] Godaux E., Cheron G., Mettens P. (1990). Ketamine induces failure of the oculomotor neural integrator in the cat. Neurosci. Lett..

[12] Mettens P., Cheron G., Godaux E. (1994). NMDA receptors are involved in temporal integration in the oculomotor system of the cat. Neuroreport.

[13] Huang Y.J., Lin C.H., Lane H.Y., Tsai G.E. (2012). NMDA Neurotransmission Dysfunction in Behavioral and Psychological Symptoms of Alzheimer’s Disease. Curr. Neuropharmacol..

[14] Puangthong U., Hsiung G.Y. (2009). Critical appraisal of the long-term impact of memantine in treatment of moderate to severe Alzheimer’s disease. Neuropsychiatr. Dis. Treat..

[15] Utine G.E., Haliloglu G., Salanci B., Cetinkaya A., Kiper P.O., Alanay Y., Aktas D., Boduroglu K., Alikasifoglu M. (2013). A Homozygous Deletion in GRID2 Causes a Human Phenotype With Cerebellar Ataxia and Atrophy. J. Child Neurol..

[16] Maier A., Klopocki E., Horn D., Tzschach A., Holm T., Meyer R., Meyer T. (2014). *De novo* partial deletion in GRID2 presenting with complicated spastic paraplegia. Muscle Nerve.

[17] Hills L.B., Masri A., Konno K., Kakegawa W., Lam A.T., Lim-Melia E., Chandy N., Hill R.S., Partlow J.N., Al-Saffar M. (2013). Deletions in GRID2 lead to a recessive syndrome of cerebellar ataxia and tonic upgaze in humans. Neurology.

[18] Van Schil K., Meire F., Karlstetter M., Bauwens M., Verdin H., Coppieters F., Scheiffert E., van Nechel C., Langmann T., Deconinck N. (2014). Early-onset autosomal recessive cerebellar ataxia associated with retinal dystrophy: New human hotfoot phenotype caused by homozygous GRID2 deletion. Genet. Med..

[19] Uebi T., Itoh Y., Hatano O., Kumagai A., Sanosaka M., Sasaki T., Sasagawa S., Doi J., Tatsumi K., Mitamura K. (2012). Involvement of SIK3 in glucose and lipid homeostasis in mice. PLOS ONE.

[20] Hemmings H.C., Yan W., Westphalen R.I., Ryan T.A. (2005). The general anesthetic isoflurane depresses synaptic vesicle exocytosis. Mol. Pharmacol..

[21] Rammes G., Danysz W., Parsons C.G. (2008). Pharmacodynamics of memantine: An update. Curr. Neuropharmacol..

[22] Seeman P., Caruso C., Lasaga M. (2008). Memantine agonist action at dopamine D2High receptors. Synapse.

[23] Lee J.W., Park H.J., Choi J., Park S.J., Kang H., Kim E.G. (2011). Comparison of ramosetron’s and ondansetron’s preventive anti-emetic effects in highly susceptible patients undergoing abdominal hysterectomy. Korean J. Anesthesiol..

[24] Khojasteh A., Sartiano G., Tapazoglou E., Lester E., Gandara D., Bernard S., Finn A. (1990). Ondansetron for the prevention of emesis induced by high-dose cisplatin. A multi-center dose-response study. Cancer.

[25] Jayadev S., Bird T.D. (2013). Hereditary ataxias: Overview. Genet. Med..

[26] Requena T., Espinosa-Sanchez J.M., Lopez-Escamez J.A. (2014). Genetics of dizziness: Cerebellar and vestibular disorders. Curr. Opin. Neurol..

[27] Rosini F., Federighi P., Pretegiani E., Piu P., Leigh R.J., Serra A., Federico A., Rufa A. (2013). Ocular-motor profile and effects of memantine in a familial form of adult cerebellar ataxia with slow saccades and square wave saccadic intrusions. PLOS ONE.

[28] Shirai Y., Asano K., Takegoshi Y., Uchiyama S., Nonobe Y., Tabata T. (2013). A simple machine vision-driven system for measuring optokinetic reflex in small animals. J. Physiol. Sci..

[29] Shutoh F., Ohki M., Kitazawa H., Itohara S., Nagao S. (2006). Memory trace of motor learning shifts transsynaptically from cerebellar cortex to nuclei for consolidation. Neuroscience.

[30] Zuo J., de Jager P.L., Takahashi K.A., Jiang W., Linden D.J., Heintz N. (1997). Neurodegeneration in Lurcher mice caused by mutation in delta2 glutamate receptor gene. Nature.

[31] Wang Y., Matsuda S., Drews V., Torashima T., Meisler M.H., Yuzaki M. (2003). A hot spot for hotfoot mutations in the gene encoding the delta2 glutamate receptor. Eur. J. Neurosci..

[32] Cull-Candy S.G., Wyllie D.J. (1991). Glutamate-receptor channels in mammalian glial cells. Ann. N. Y. Acad. Sci..

[33] Kato A.S., Knierman M.D., Siuda E.R., Isaac J.T., Nisenbaum E.S., Bredt D.S. (2012). Glutamate receptor delta2 associates with metabotropic glutamate receptor 1 (mGluR1), protein kinase Cgamma, and canonical transient receptor potential 3 and regulates mGluR1-mediated synaptic transmission in cerebellar Purkinje neurons. J. Neurosci..

[34] Hirai H., Launey T., Mikawa S., Torashima T., Yanagihara D., Kasaura T., Miyamoto A., Yuzaki M. (2003). New role of delta2-glutamate receptors in AMPA receptor trafficking and cerebellar function. Nat. Neurosci..

[35] Yamasaki M., Miyazaki T., Azechi H., Abe M., Natsume R., Hagiwara T., Aiba A., Mishina M., Sakimura K., Watanabe M. (2011). Glutamate receptor delta2 is essential for input pathway-dependent regulation of synaptic AMPAR contents in cerebellar Purkinje cells. J. Neurosci..

[36] Honore T., Davies S.N., Drejer J., Fletcher E.J., Jacobsen P., Lodge D., Nielsen F.E. (1988). Quinoxalinediones: Potent competitive non-NMDA glutamate receptor antagonists. Science.

[37] Kakegawa W., Miyazaki T., Kohda K., Matsuda K., Emi K., Motohashi J., Watanabe M., Yuzaki M. (2009). The N-terminal domain of GluD2 (GluRdelta2) recruits presynaptic terminals and regulates synaptogenesis in the cerebellum *in vivo*. J. Neurosci..

[38] Rabacchi S., Bailly Y., Delhaye-Bouchaud N., Mariani J. (1992). Involvement of the N-methyl d-aspartate (NMDA) receptor in synapse elimination during cerebellar development. Science.

[39] Garthwaite J., Brodbelt A.R. (1989). Synaptic activation of N-methyl-d-aspartate and non-N-methyl-d-aspartate receptors in the mossy fibre pathway in adult and immature rat cerebellar slices. Neuroscience.

[40] Yuzaki M., Forrest D., Verselis L.M., Sun S.C., Curran T., Connor J.A. (1996). Functional NMDA receptors are transiently active and support the survival of Purkinje cells in culture. J. Neurosci..

[41] Balazs R., Jorgensen O.S., Hack N. (1988). N-Methyl-d-aspartate promotes the survival of cerebellar granule cells in culture. Neuroscience.

[42] Ortega F., Perez-Sen R., Morente V., Delicado E.G., Miras-Portugal M.T. (2010). P2X7, NMDA and BDNF receptors converge on GSK3 phosphorylation and cooperate to promote survival in cerebellar granule neurons. Cell. Mol. Life Sci..

[43] Kadotani H., Hirano T., Masugi M., Nakamura K., Nakao K., Katsuki M., Nakanishi S. (1996). Motor discoordination results from combined gene disruption of the NMDA receptor NR2A and NR2C subunits, but not from single disruption of the NR2A or NR2C subunit. J. Neurosci..

[44] Marmolino D., Manto M. (2010). Past, present and future therapeutics for cerebellar ataxias. Curr. Neuropharmacol..

[45] Bormann J. (1989). Memantine is a potent blocker of N-methyl-d-aspartate (NMDA) receptor channels. Eur. J. Pharmacol..

[46] Strupp M., Brandt T. (2009). Current treatment of vestibular, ocular motor disorders and nystagmus. Ther. Adv. Neurol. Disord..

[47] Yoshida T., Katoh A., Ohtsuki G., Mishina M., Hirano T. (2004). Oscillating Purkinje neuron activity causing involuntary eye movement in a mutant mouse deficient in the glutamate receptor delta2 subunit. J. Neurosci..

[48] Faulstich M., van Alphen A.M., Luo C., du Lac S., De Zeeuw C.I. (2006). Oculomotor plasticity during vestibular compensation does not depend on cerebellar LTD. J. Neurophysiol..

[49] Gordon J.W., Uehlinger J., Dayani N., Talansky B.E., Gordon M., Rudomen G.S., Neumann P.E. (1990). Analysis of the hotfoot (ho) locus by creation of an insertional mutation in a transgenic mouse. Dev. Biol..

[50] Kashiwabuchi N., Ikeda K., Araki K., Hirano T., Shibuki K., Takayama C., Inoue Y., Kutsuwada T., Yagi T., Kang Y. (1995). Impairment of motor coordination, Purkinje cell synapse formation, and cerebellar long-term depression in GluR delta 2 mutant mice. Cell.

[51] Wilson D.B. (1975). Brain abnormalities in the lurcher (Lc) mutant mouse. Experientia.

[52] Nishiyama J., Matsuda K., Kakegawa W., Yamada N., Motohashi J., Mizushima N., Yuzaki M. (2010). Reevaluation of neurodegeneration in lurcher mice: Constitutive ion fluxes cause cell death with, not by, autophagy. J. Neurosci..

[53] Cendelin J., Tuma J., Korelusova I., Vozeh F. (2014). The effect of genetic background on behavioral manifestation of Grid2(Lc) mutation. Behav. Brain Res..

[54] Schwartz E.J., Rothman J.S., Dugue G.P., Diana M., Rousseau C., Silver R.A., Dieudonne S. (2012). NMDA receptors with incomplete Mg(2)(+) block enable low-frequency transmission through the cerebellar cortex. J. Neurosci..

[55] Hepp R., Hay Y.A., Aguado C., Lujan R., Dauphinot L., Potier M.C., Nomura S., Poirel O., El Mestikawy S., Lambolez B. (2014). Glutamate receptors of the delta family are widely expressed in the adult brain. Brain Struct. Funct..

[56] Glitsch M.D. (2008). Calcium influx through N-methyl-d-aspartate receptors triggers GABA release at interneuron-Purkinje cell synapse in rat cerebellum. Neuroscience.

[57] Liu S.J. (2007). Biphasic modulation of GABA release from stellate cells by glutamatergic receptor subtypes. J. Neurophysiol..

[58] Duguid I.C., Smart T.G. (2004). Retrograde activation of presynaptic NMDA receptors enhances GABA release at cerebellar interneuron-Purkinje cell synapses. Nat. Neurosci..

[59] Hirano T., Watanabe D., Kawaguchi S.Y., Pastan I., Nakanishi S. (2002). Roles of inhibitory interneurons in the cerebellar cortex. Ann. N. Y. Acad. Sci..

[60] Whittingham D.G. (1968). Fertilization of mouse eggs *in vitro*. Nature.

[61] Tabata T., Sawada S., Araki K., Bono Y., Furuya S., Kano M. (2000). A reliable method for culture of dissociated mouse cerebellar cells enriched for Purkinje neurons. J. Neurosci. Methods.

